# Gene Therapy for Fragile X Syndrome, Challenges, and Promises

**DOI:** 10.1002/jgm.70049

**Published:** 2025-11-09

**Authors:** Milen Velinov

**Affiliations:** ^1^ Rutgers Robert Wood Johnson Medical School New Brunswick New Jersey USA

**Keywords:** *FMR1*, FMRP, fragile X syndrome, gene therapy

## Abstract

Fragile X syndrome (FXS) is the most common single gene cause of inherited intellectual disability and autism spectrum disorder (ASD). *FMR1*, the gene associated with FXS, is located on chromosome X. Accordingly, males with loss‐of‐function (full) mutations are more severely affected than females. Strategies for therapeutic intervention for this disorder have included behavioral and medication therapy. To date, no management strategies have been shown to be curative. Gene therapy that aims to supply the functional protein product of the gene *FMR1* to the brain is an attractive concept for curative treatment. Experiments aimed at activating the mutant *FMR1*, modifying its abnormal RNA product, or supplying functional *FMR1* copies to the CNS have been conducted. The delivery of the FMR1 gene and its product to animal models of FXS have been primarily conducted with intrathecal applications because of the low efficiency of the gene therapy vectors to cross the blood–brain barrier (BBB). This delivery approach is associated with a higher risk of complications and appears to distribute the gene product unevenly across different brain regions. We have explored the efficiency of a recently developed adeno‐associated virus (AAV) vector with increased BBB crossing in certain strains of mice to deliver *FMR1* with peripheral IV administration. Our experiments demonstrated very high delivery efficiency and also highlighted the risk of oversupplying the brain with FMRP, the protein product of the *FMR1* gene. Other AAV vectors with enhanced crossing of the BBB in primates have been developed, providing an attractive option for further experiments involving peripheral administration. Providing the gene product to specific brain cells remains a difficult challenge for future experiments. It may also be important or even necessary to regulate the gene expression to mimic physiological expression patterns since the levels of FMRP change dramatically during development, with maximum levels early in the postnatal period and a decline across early life. In addition, there are 12 identified mouse isoforms of FMRP due to alternative RNA splicing, and an even higher number of isoforms is found in humans. It may thus be a challenge to determine what *FMR1* isoform or set of isoforms would have the optimum efficiency in correcting the phenotype. Despite these challenges, the recent developments establish the basis for future research to develop efficient and minimally invasive gene therapy protocols for FXS.

## Graphical abstract

Supplying the functional protein product of the *FMR1* gene to the brain is an attractive concept for curative treatment of Fragile X syndrome. Despite existing challenges, recent developments establish the basis for developing efficient gene therapy protocols for this condition.

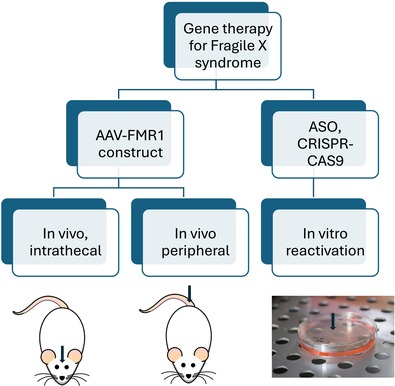

## 1 The Molecular Basis of Fragile X Syndrome

Fragile X syndrome (FXS) is a neurodevelopmental disorder resulting from an X‐linked mutation in *FMR1*. The overall prevalence of FXS is approximately 1 in 7000 in males and 1 in 11,000 in females [1, 2, 3]. In almost all cases, the FXS phenotype is caused by an expansion of more than 200 repeats and the subsequent methylation of CGG triplets in the 5′ untranslated region of the *FMR1* gene [3]. All FXS cases are associated with absent or markedly decreased production of the *FMR1* protein product, the fragile X messenger ribonucleoprotein (FMRP) (formerly, fragile X mental retardation protein). FMRP is highly conserved among vertebrates and functions as an mRNA‐binding protein that regulates the localization, stabilization, and translation of approximately 4% of fetal brain mRNA [4, 5, 6, 7, 8]. FMRP regulates the translation of many synaptic proteins involved in high‐fidelity information processing at the synapse [9]. FMRP is also known to associate with the mRNAs of many genes associated with autism spectrum disorder (ASD) and Intellectual Disability (ID) [10]. The extensive role of FMRP in translational regulation in the brain is evolutionarily conserved as demonstrated in studies of *Drosophila* oocytes and the mouse cortex [11]. There are at least 12 identified mouse isoforms of the FMRP protein that are products of alternative mRNA splicing [12]. The level of expression of each isoform varies during development and modulates binding affinities to ribosomes. FMRP is diffusely expressed throughout the brain, and its levels vary widely among subpopulations of neurons [13]. Based on this and previous studies, FMRP levels vary largely across brain regions. The highest expression is in the olfactory bulb, isocortex, hippocampus, thalamus, and cerebellum. In contrast, FMRP levels are generally low in the hypothalamus and midbrain. In neurons, FMRP localizes to the soma, axon, and dendritic compartments [3, 13]. The overall expression of FMRP changes dramatically during development, typically peaking in early development and declining across early life [3, 14, 15]. Interestingly, different FMRP isoforms exhibit distinct subcellular localizations, suggesting that these isoforms may have diverse, independent functions, and their loss may contribute to the FXS phenotype through multiple pathophysiological pathways [16, 17]. The most prevalent form of FMRP in humans contains 632 amino acids and is a classic RNA‐binding protein containing at least three canonical RNA‐binding motifs: two hnRNP K homology (KH) domains and an arginine‐glycine–glycine (RGG) box [18]. In a recent study, a group from the University of Massachusetts and Rush University showed widespread RNA mis‐splicing in peripheral tissues of individuals with FXS [19]. Interestingly, this study discovered an aberrant *FMR1*‐RNA expression in a mosaic manner in individuals with FXS. This aberrant RNA, also referred to as RNA‐217, is the product of mis‐splicing and includes exon 1 and a pseudo‐exon out of intron 1 of *FMR1*. This discovery contradicted the previously maintained belief that the full mutation *FMR1* is completely silenced. The discovery opened an opportunity to use antisense oligonucleotides (ASOs) to block this RNA mis‐splicing and restore the FMRP synthesis (see Section 8.4).

## 2 The Clinical Manifestations of FXS

Males with a full *FMR1* mutation present with the features typically associated with FXS [19]. The body mass index (BMI) of individuals with FXS is significantly higher compared to the normative reference at all ages and among both males and females, and this disparity increases with age. In contrast, the height percentile decreases with age and is significantly lower than in the normative reference by adulthood [20]. All early milestones, including speech, gross motor, and fine motor skills, are delayed [19] The IQ scores are typically in the moderate intellectual disability range [21]. All full mutation males and some females have manifestations of abnormal behaviors, including repetitive, stereotypical movements, hyperactivity, anxiety, and decreased eye contact. Some patients show aggressive and self‐injurious behaviors. These behavioral manifestations tend to increase with age [22]. These behavioral characteristics are disabling and interfere with the patient's management [23]. The phenotype of FXS also involves poor eye contact, excessive shyness, aggression, tactile defensiveness, impulsivity, hyperarousal to sensory stimuli, and ASD [21]. More than half of the males and nearly 20% of females meet the *Diagnostic and Statistical Manual of Mental Disorders, Fifth Edition* criteria for ASD [24]. FXS is the most common single‐gene defect identified in patients with ASD. Accordingly, diagnostic testing for FXS is recommended as a first‐tier genetic test in current expert guidelines for the management of ASD [25].

## 3 The Social Significance of FXS

The cost of caring for patients with FXS has been estimated in several studies and appears to vary significantly across different countries. In a study estimating the direct cost of the care for the US patients with FXS compared to non‐FXS controls for the period 1997–2012, the patients with FXS had an incremental annual total healthcare cost of $33,409 (2012) per person relative to the comparison group, while newly diagnosed FXS patients had incremental total annual healthcare costs of $17,617 (2012) per person [26]. This study estimated the direct cost of healthcare. Ideally, the cost estimate for the FXS families should also include lost family income associated with the need to care for the patients. Given the relatively high prevalence of this condition, the cumulative economic cost for the care of FXS is significant.

## 4 Mosaicism in FXS

### 4.1 Size and Methylation Mosaicism

Because of the instability of the CGG repeat during transmission, individuals can be comprised of a mixture of cells in which a proportion have a normal or premutation *FMR1* allele and the remaining cells have a full‐mutation allele. FMRP production only occurs in those cells with the normal or premutation allele [27]. In addition, the *FMR1* silencing in full mutation alleles through gene methylation may not occur equally in all cells. This may result in a second type of *FMR1* mosaicism: methylation mosaicism, in which the full‐mutation allele escapes methylation and can produce FMRP in a variable proportion of cells [27, 28]. A study of a large number of mosaic full mutation FXS individuals showed that the patients' IQ corresponds to the degree of methylation mosaicism but not to the degree of size mosaicism [29].

## 5 The Correlation of FMRP Levels With Cognitive Functioning

In a recent study, FMRP levels were correlated with IQ in individuals with FXS and normal controls [30]. The authors concluded that there is a threshold level of FMRP at approximately 70% (−1 SD) of the mean FMRP level for those with normal CGG‐repeat alleles, above which there is no further dependence of IQ on FMRP levels—that is, a potential state of sufficiency such that further increases above the threshold do not relate to further cognitive gain. Even more importantly, this study confirmed previous evidence from animal studies that an FMRP level of only 10%–20% of normal would support a mean IQ at the borderline level.

In relation to gene therapy, this finding suggests that delivering only 20% of functioning FMR1 may be sufficient to significantly improve the intellectual functioning in FXS.

## 6 Current Management Strategies for FXS

### 6.1 Nonmedication Therapies

Currently, the management of individuals with FXS is supportive and targets specific manifestations associated with the disorder. Referrals to early intervention programs for patients younger than 3 years or to school‐assisted Individual Education Plans for those 3 years or older are a milestone in the current management in the United States. Patients with FXS benefit from help with gross and fine motor development, communication, and abnormal behaviors [19, 31]. Behavioral and educational interventions are helpful in individuals with FXS [32]. Such interventions may include applied behavior analysis and speech and language therapy [24, 33]. Such approaches of behavioral therapy may help address challenging behaviors, improve communication skills and language development, and teach social and adaptive skills. Occupational and physical therapy can enhance fine motor skills, sensory processing, and daily activities of living [33, 34].

### 6.2 Medication Therapies

Medications are used to treat symptoms in FXS patients similarly to non‐FXS patients. FXS patients tend to be more sensitive to psychoactive medications and may need lower initial doses. Psychostimulants (such as methylphenidate‐ or dextroamphetamine‐based medications), atomoxetine (a selective norepinephrine reuptake inhibitor), alpha‐agonists, and alternative pharmacologic treatments (folic or folinic acid, L‐acetyl carnitine) are often used for ADHD manifestations. Selective serotonin reuptake inhibitors (SSRIs) and antipsychotic medications are used to treat anxiety, irritability, and aggressive behaviors. Sleep problems may be controlled with melatonin, clonidine, guanfacine, trazodone, and quetiapine [19].

There are multiple past and current clinical trials for medications that attempt to address specific downstream consequences of the FMRP deficit. For instance, metformin, cannabidiol, BPN14770 (a phosphodiesterase‐4D inhibitor), and minocycline have shown positive results in pilot clinical trials for controlling certain aspects of the patient's behavior and even improving cognitive skills [31]. However, to date, there is no FDA‐approved medication specifically for FXS. FMRP has a widespread effect on multiple brain functions [3]. Accordingly, it would be practically impossible to address all resulting clinical manifestations with a medication that targets a specific downstream deficiency. The status of existing treatments for FXS makes the gene therapy approach more attractive since the supply of a functioning *FMR1*, at least in theory, promises to address all deficiencies.

## 7 Gene Therapy—Definitions and Approaches

Gene therapies generally refer to the correction of abnormal gene function using nucleic acids [35]. Some therapy protocols that utilize small, nonnucleic acid molecules may also be considered part of this group of treatments, as they directly address deficient genetic functions [36, 37]. The approaches of gene therapy differ by the type of correction and the type of therapy agent.

## 8 Gene Therapy Trials for FXS

Soon after the elucidation of the molecular basis of FXS, the concept of gene therapy for the condition was suggested as a viable option [38]. FXS is a disorder that primarily affects brain function. Therefore, a major obstacle has been and remains the difficulty of delivering the gene therapy agent across the blood–brain barrier (BBB).

### 8.1 Animal Models for FXS

Efforts to develop mouse models for FXS using a knock out (KO) strategy started soon after the elucidation of its molecular basis [39]. The subsequently developed KO models recaptured the FMRP deficiency and showed both behavioral and physical similarities with FXS in humans [40, 41, 42, 43]. However, the KO models do not recapture the molecular basis of FXS in humans since they do not include the 5′ repeat expansion. Efforts were made to develop (knock in) KI repeat expansion‐based models that would better recapture the molecular defect in humans. However, Fmr1 in these expanded repeat mouse models does not become methylated, and the FMRP production is intact, unlike in humans [44, 45]. This difference between human and mouse biology makes it challenging to use animal disease models for *FMR1* reactivation studies. The lack of animal models for FXS that follow the same molecular pathways fueled studies on human cell lines as an alternative, more relevant model [46]. Human FXS cell lines are currently used to conduct *FMR1* reactivation as a strategy for gene therapy (see sections 8.4 and 8.5).

### 8.2 Direct Intrathecal Injection of FMR1 Carrying Vector

In 2009, a group from the University of Florida treated FMR1 KO mice with an adeno‐associated virus (AAV) construct that includes the major isoform of *FMR1* [47]. The promoter was the chicken β‐actin core promoter. The gene construct was injected bilaterally into the hippocampus. The injected vector restored FMRP expression in the hippocampus of KO mice and rescued the phenotype of a characteristic enhanced long‐term depression in the treated KO mouse hippocampus [47]. In 2014, a group at the University of Toronto reported the results of an intracerebroventricular administration of an AAV serotype 9 construct that included the prevalent isoform 1 of *FMR1*. The gene construct had a neuron‐specific synapsin I promoter. About 50% of the normal FMRP levels were observed after injection of the construct in the cortex and the hippocampus of *Fmr1* KO mice [48]. In this study, FMRP was not detected in brain regions more distal from the lateral ventricles, which were located too far from the ventricles to acquire sufficient vector uptake. Rescue or partial rescue of stereotypic behaviors (marble burying test) and deficit in social dominance (tube test), but not of motor hyperactivity or incidence of audiogenic seizures, were observed in the treated KO animals [48]. In 2021, the same group studied the effect of intracerebroventricular and intracisternal magna injection of an AAV9 vector‐based construct that had mouse isoform 7 and rat isoform X3 with a mini‐MeCP2 promoter in KO mice and rat models, respectively. Mouse isoform 7 and rat isoform X3 are orthologs of the human isoform 17, which has the highest transcript levels in the brain [49]. Postnatal treatment resulted in partial rescue of the Fmr1 KO rat phenotype, including improved social dominance in treated *Fmr1* KO females and partial rescue of locomotor activity in males. Electroencephalogram (EEG) recordings showed correction of abnormal slow‐wave activities during the sleep‐like state in male Fmr1 KO rats. For both rodent species, expression was high in the motor and frontal cortices and moderate in the hippocampus and in the striatum of the rat and the thalamus of the mouse. The transgene expression was more limited in the caudal regions of the brain, including the cerebellum and brainstem [49]. In a more recent study, the group injected intrathecally the human isoform 17 of *FMR1* in an AAV vector and observed significant levels of FMRP in various brain regions, including the cortex and the hippocampus. This increased expression persisted when measured at 1, 3, and 6 months after the treatment [50] Improvement in seizure control and fear conditioning was observed in the treated KO mice.

### 8.3 Peripheral IV Gene Therapy

**FIGURE 1:**
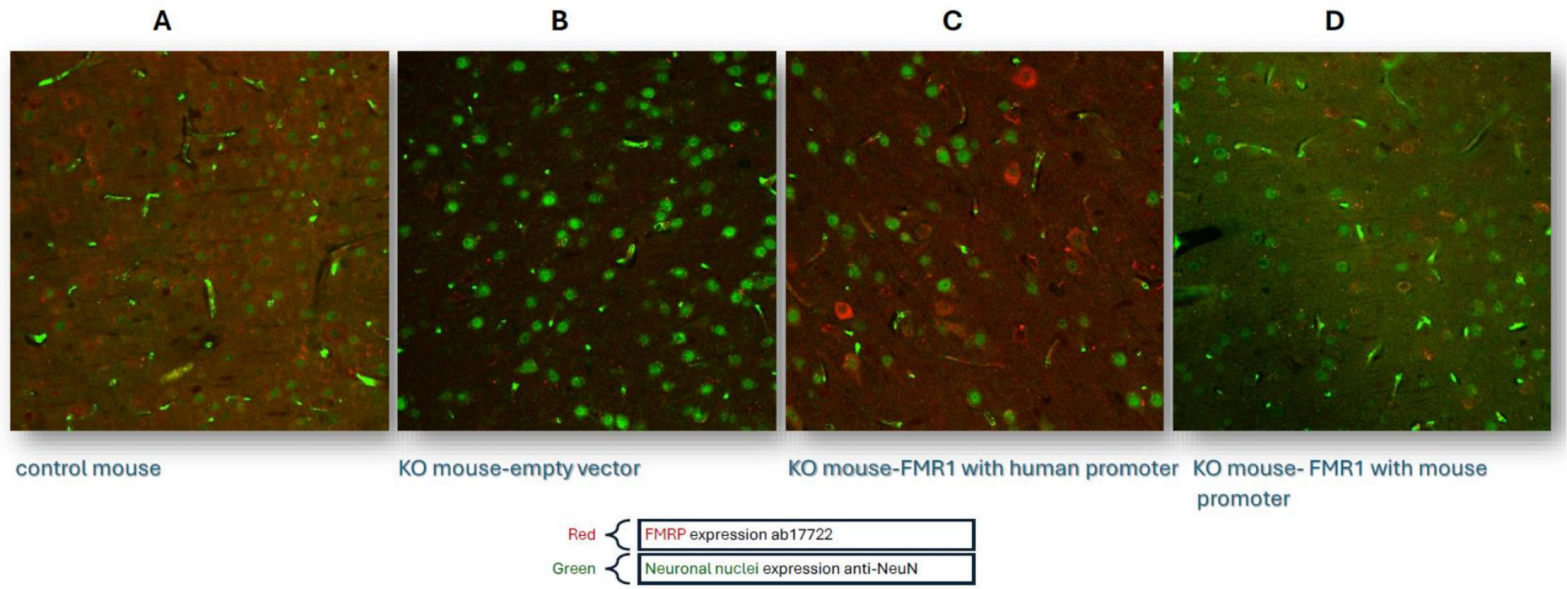
Mouse neocortex at 40 X (*n* = 1): Efficient delivery of FMRP in mouse neonatal cortex after peripheral administration of AVV gene construct (adopted with slight modifications from [55].

Peripheral administration of *FMR1‐containing* vectors may help reduce therapy complications associated with the method of delivery and provide a more even distribution of the transgene across different brain regions. There is substantial research aimed at developing AAV9 vectors with higher efficiency in crossing the BBB, thereby allowing for the peripheral application of gene therapy treatments for CNS disorders [51]. One more traditional AAV9 vector was successfully used to develop a treatment for spinal muscular atrophy (SMA) and is currently FDA‐approved [52]. We took advantage of the availability of vector AAV.php, which has shown very high efficiency in crossing the BBB in certain mouse strains [53]. We used an AAV.php vector that includes the mouse *Fmr1* isoform 7 gene with a human synapsin promoter, injected into the tail vein of KO and control mice [54]. A 2 × 10^13^ vector genome per kilogram, vg/kg, was administered in 4‐week‐old mice. Robust transgene expression, producing FMRP levels higher than in wild‐type mice, was observed in KO mice in all brain regions but not outside the brain. The treated KO mice showed correction of deficits on the rotarod assay. The treatment also partially improved the animals' performance on the fear conditioning assays [54]. Efficient and evenly distributed FMRP delivery in the mouse neocortex in our study is shown in Figure 1. When correlations were made for individual mice between FMRP levels and their rotarod performance, surprisingly, the FMRP levels inversely correlated with the rotarod performance, suggesting that FMRP oversupply may interfere negatively with brain functioning. In support of these findings, FMRP duplications were reported in association with intellectual disability in humans [56, 57].

### 8.4 Gene Therapy for FXS by Reactivating the FMR1 Gene

Since *FMR1* in FXS is deactivated by methylation, a study of FXS cell lines explored the possibility of reactivating the gene with the use of a hypomethylating agent. In one of the earlier studies of *FMR1* de‐methylation using 5‐azadeoxycytidine (5‐azadC) in fragile X patients' lymphoblastoid cells, the authors showed in vitro *FMR1* reactivation and FMRP production [58]. In another more recent study, in 5‐azadC‐treated FXS‐Neuronal Progenitor Cells, the authors showed an *FMR1* mRNA level equivalent to 40% of that in normal cells following 6 days of treatment. The mRNA levels increased to a maximum of 60%–70% of normal cells 6 days after the drug was removed. The treated cells did not show any decline in *FMR1* expression during the entire culturing period following 5‐azadC withdrawal, as was shown by real‐time PCR analyses 30 days after the end of the treatment [59]. Further, systemic treatment with a DNA Methyltransferase (DNMT) inhibitor in mice carrying FXS‐induced pluripotent stem cell (iPSC)‐derived transplants robustly induced *FMR1* expression in the affected tissue, which was maintained for a prolonged period. Similarly, another group showed reactivation of *FMR1* in fibroblast and lymphoblastoid cell lines from patients with FXS using the hypomethylating agent chaetocin used in combination with 5‐azadeoxycytidine. Additionally, chaetocin, BIX01294, and 3‐Deazaneplanocin A (DZNep) were able to delay the re‐silencing of 5‐azadeoxycytidine reactivated FMR1 alleles significantly [60]. Hypomethylating agents are commonly used as anticancer drugs, but their use is limited due to their low tolerability [61]. The unspecific demethylation approach has major limitations for human use because of the toxicity of the demethylating agents. Using non–gene‐specific demethylating agents may lead to significant side effects related to the demethylation of other unrelated genomic regions. Finally, no suitable animal model exists for applying this technology because in the available models, the *FMR1* is not inactivated via methylation of trinucleotide repeats.

Clustered regularly interspaced short palindromic repeats (CRISPR) and CRISPR‐associated protein 9 (Cas9) technology was also used to selectively reactivate *FMR1* by truncating the CGG repeats without affecting other genes or by targeted de‐methylation [62, 63, 64]. The authors of the most recent such study [63] had previously fused a catalytically inactive Cas9 with the DNA methylation modification enzymes Dnmt or Tet (dCas9‐Dnmt/Tet), allowing for targeted modification of DNA methylation in the mammalian genome. They have also developed a single sgRNA to guide the dCas9‐Tet1 to the CGG repeat region of FMR1. They were able to restore FMRP expression in FXS iPSCs and differentiated neurons with no significant off‐targeting effect. Furthermore, they were able to demonstrate in vivo FMRP expression in engrafted modified human cells in mouse brains [64].

As mentioned in Section 1, a recent study showed that, surprisingly, 70% of the cells of affected FXS individuals generate an aberrant RNA transcript of the mutant *FMR1 gene* [65]. A previously unknown RNA isoform referred to as FMR1 217 RNA is generated as a result of mis‐splicing. The authors used ASOs to block this RNA missplicing to produce the correct protein product of FMRP. They showed that treating the cells with mis‐spliced FMR1 RNA with ASOs may reverse the missplicing and generate a normal FMRP product [65]. Such a strategy was previously used for the management of SMA and was FDA‐approved [66].

ASO‐ and CRISPR‐based strategies have some advantages compared to AAV gene delivery since they may potentially reactivate the expression of all *FMR1* isoforms, something that is not possible to achieve using AAV‐mediated delivery. In addition, based on current experience with other disorders, the ASO treatments seem to have lower risks for complications compared to AAV delivery protocols. A major disadvantage of the ASO molecules is that these agents target RNA molecules and therefore require repeated administration. At this time, ASO and CRISPR agents have low efficiency in crossing the BBB and are typically administered intrathecally. Recently reported facilitated BBB crossing of ASO‐Apo E conjugates may help achieve higher BBB crossing efficiency [67]. Finally, the lack of appropriate animal models makes it difficult to assess the clinical effects of these treatments.

The reported to‐date approaches for gene therapy in FXS and the way of delivery are summarized in Table 1

**TABLE 1:** Summary of the discussed publications reporting FXS gene therapy studies.

Year	Reference	Agent	Promoter	Disease model	Delivery	Results
	Gene delivery					
2009	49	AAV‐FMR1 (major isoform)	Chicken ß‐actin core promoter	KO mice	Intrathecal	Rescued long‐term‐depression in hippocampus
2014	50	AAV‐FMR1 (isoform 1)	Synapsin I	KO mice	Intrathecal	Rescue of stereotypic behaviors and deficit in social dominance
2021	51	AAV‐FMR1 (mouse isoform 7 and rat isoform X3)	Mini‐MECP2 promoter	KO mice and rats	Intrathecal	Improved social dominance and correction in EEG abnormalities in rats
2023	52	AAV‐FMR1‐human isoform 17	JeT recombinant promoter	KO mice	Intrathecal	Improved seizure control and fear conditioning
2023	56	AAV‐FMR1‐mouse isoform 7	Synapsin I	KO mice	Tail vein	Correction of the deficits on rotarod assay and fear conditioning

	Gene reactivation		Strategy			
1998	59	5‐azadeoxycytidine	FMR1 demethylation	Lymphoblastoid cell lines	Direct treatment	Restored FMR1 production
2019	60	5‐azadeoxycytidine	FMR1 demethylation	FXS‐neuronal progenitor cells	Direct treatment	Restored 60%–70% of FMR1 RNA levels
2020	61	Chaetocin, BIX01294 and 3‐Deazaneplanocin A	FMR1 demethylation	FXS lymphoblastoid lines	Direct treatment	Delayed re‐silencing of FMR1 after reactivation
2015	63	CRISPR‐Cas9	Removal of the CGG repeats	FXS‐iPSC	Transfection	FMR1 reactivation was retained through neural differentiation
2016	64	CRISPR‐Cas9	Removal of the CGG repeats	FXS iPS cells	Transfection	Sustained FMR1 reactivation for 50 days
2018	65	CRISPR‐Cas9	Targeted demethylation of the FMR1 CGG repeats	FXS iPS cells and neuronal cells	Transfection	Sustained FMR1 reactivation including in cell engrafts in research animals
2023	19	ASO + 5‐azadeoxycytidine	Blocking an aberrant FMR1 RNA and demethylation	FXS lymphoblastoid cell lines	Transfection	Partial restoration of FMR1 expression

## 9 Challenges

### 9.1 BBB Crossing

The low efficiency of transferring nucleic acids across the BBB remains an essential challenge for the gene therapy of FXS. In our experiments, we achieved very high efficiency of brain delivery using an AAV construct [54]. While the use of the AVA.php vector in our experiments led to impressively high efficiency of the *FMR1* delivery, such experiments may be successful only in certain strains of mice and are not applicable for use in humans. This vector uses a mouse‐specific LY6A (SCA1) receptor that enhances the BBB crossing [54, 68, 69]. Overcoming the low BBB crossing efficiency may be achieved by using recently developed AAV vectors that have demonstrated high LY6A‐independent BBB crossing in mice and primates [70].

### 9.2 Choice of Promoters

Most studies on gene therapy for FXS have utilized CNS‐specific promoters. In cases of peripheral applications, such brain‐specific promoters are especially important, as the absence of FMRP outside the CNS may help prevent an immune reaction against the *FMR1* transgene product in FXS individuals who have not encountered FMRP in their previous life. Anti‐transgene immune reactions have been previously observed in gene therapy for Duchenne muscular dystrophy (DMD) [71]. One potential problem with CNS‐specific promoters for applications in humans is the inability to monitor the FMRP levels in peripheral blood. Therefore, it seems that efforts should be made to identify peripheral downstream markers with levels corresponding to the FMRP levels in the brain.

### 9.3 Timing and Level of FMRP Supply

Another potential problem may be the significant changes in the FMRP levels during the lifetime [3, 14, 15]. This may lead to an FMRP overdose when the necessary physiological levels may be lower than the transgene generated. One possible way to avoid such complications is to use a regulable promoter [72]. Alternatively, FMRP may be delivered in a lower dose compared to physiological levels. As previously mentioned, it was shown that only 10%–20% of the physiological FMRP level would support a borderline average cognitive functioning [30].

### 9.4 Early Applications

In our experiments, we administered the FMR1 construct to 4‐week‐old mice, which is the equivalent of young adults in humans. The optimal age for administering the gene therapy product is at an earlier age, ideally soon after birth. However, access to the mouse tail vein used in our experiments may be more difficult in younger mice. A possible alternative may be to use the mouse facial vein that is accessible in newborn mice. Preclinical trials with application via the facial vein were previously used for SMA [73]. Such an access issue would not be relevant for application in humans. A prerequisite for early human applications would be early diagnosis. Currently, carrier screening for FXS is recommended in guidelines by the American College of Medical Genetics [74]. However, at this time, FXS is not included in the recommended universal newborn screening panel [75]. An available efficient treatment is a prerequisite to include a specific condition in the newborn screening programs. In addition, the minimal signs of FXS in early life may result in the unwillingness of the family to consent to a risk associated with treatment.

### 9.5 FMR1 Isoforms‐Treatment With Infusion of Several Isoforms

The AAV constructs have limited capacity for transgene incorporation. Therefore, until now, many gene therapy trials for FXS have been done with one specific isoform of *FMR1*. Past studies have preferentially used the mouse isoform 7, which is an ortholog of the human isoform 17 and is the most abundantly expressed in the brain. However, as previously mentioned, the expression level of each isoform varies during development and modulates binding affinities to ribosomes. FMRP is diffusely expressed throughout the brain and varies widely among subpopulations of neurons [13]. In a previous study, AAV constructs carrying two different FMR1 isoforms were directly administered to the brains of KO mice. These experiments resulted in improvements in social deficits and repetitive behaviors in an animal model. Human *FMR1* promoter was used in these experiments [76].

## 10 Conclusions

One important aspect of the future development of gene therapy for FXS is the use of viral vectors with higher efficiency for crossing the BBB. Such vectors are in the pipeline and will soon be available for use in humans. However, the future development of gene therapy approaches for these patients may also be defined by disease severity. Unlike SMA and DMD, disorders for which gene therapy protocols were quickly accepted by the patients' families, FXS does not lead to a significant decrease in life span. While the management of FXS patients is often very challenging, many of these patients may have a normal life span. AAV‐based gene therapy is not without risks. Cases of fatal complications, while rare, have been reported in patients with SMA and DMD who received gene therapy [77, 78]. Fatal outcomes may be related to liver failure because of anti‐viral immunity. Families of FXS patients and their treating physicians may have higher safety requirements for such protocols compared to the other groups. While the efficiency of crossing the BBB may be significantly improved with the development of new AAV vectors, the risk of liver damage may not be easy to reduce. Thus, nonviral gene carriers may become the leading approach in the future [79]. One major obstacle for the future development of *FMR1* gene re‐activation may be the lack of a suitable animal model. Current mouse models of FXS are developed with *FMR1* knockout strategies and do not include repeat expansion‐associated methylation and gene inactivation. It may also be difficult to lower the risks of significant adverse effects since these methods are associated with risk for nonspecific effects of the de‐methylating agents, as seen in chemotherapy protocols [61]. The remaining challenge is also the identification of the most efficient *FMR1* isoform or set of isoforms. This is likely to be addressed with the use of different isoform combinations. Lastly, the fluctuation of FMRP levels during early life and the risk for overdosing may require starting therapy protocols with a lower dose of the gene, which may be with lower efficiency. The *FMR1* reactivation or CRISPR‐based strategies may help provide physiologic isoform expression and appropriate FMRP levels during development. Monitoring of the FMRP levels after administration of the gene therapy agent may also be a challenge if CNS‐specific promoters are used since the level of FMRP outside of the CNS will be very low or absent. Despite these challenges, gene therapy for FXS seems to be the most promising approach in the long term for the cure of the disorder since it has the potential to address multiple downstream effects of the FMRP deficiency. This innovative approach is becoming an exciting field with likely breakthrough developments in the near future. No IRB approval was required for this study.

## Author Contributions

M.V. reviewed the published data and wrote the manuscript.

## Conflicts of Interest

The author declares no conflicts of interest.

## Data Availability

Data are available upon request.
